# Retraction: CRISPR/Cas9-Mediated OC-2 Editing Inhibits the Tumor Growth and Angiogenesis of Ovarian Cancer

**DOI:** 10.3389/fonc.2021.694036

**Published:** 2021-05-10

**Authors:** Frontiers Editorial Office

**Affiliations:** Frontiers Media SA, Lausanne, Switzerland

The journal retracts the September 2, 2020 article cited above for the following reasons:

Following publication, concerns were raised regarding manipulation in **Figures 5** and **7**. An investigation was conducted in accordance with Frontiers’ guidelines and the authors failed to provide a satisfactory explanation.

This retraction was approved by the Chief Editors of Frontiers in Oncology and the Editor-in-Chief of Frontiers. The authors agree to this retraction.

